# Evaluation of right ventricular function in fetal hypoplastic left heart syndrome using spatio-temporal image correlation (STIC)

**DOI:** 10.1186/s12947-016-0056-5

**Published:** 2016-04-11

**Authors:** Jing Zhang, Qichang Zhou, Yili Zhao, Qinghai Peng, Zheli Gong, Xiangdang Long

**Affiliations:** 1 Department of Ultrasound, Second Xiangya Hospital of Central South University, 139 Middle Renmin Rd, 410011 Changsha, Hunan China; 2 Department of Obstetrics and Gynecology, Eastern Virginia Medical School, 825 Fairfax Avenue, Norfolk, 23507 VA USA

**Keywords:** Fetal hypoplastic left heart syndrome, Right ventricular function, Spatio-temporal image correlation

## Abstract

**Background:**

Postnatal outcome of fetuses with hypoplastic left heart syndrome (HLHS) is mainly determined by right ventricular function. In the present study we used spatio-temporal image correlation (STIC) to assess right ventricular function of fetuses with HLHS.

**Methods:**

Three-dimensional ultrasound with STIC technique was used to acquire heart images from fetuses that had HLHS and the normal controls, between 24^+0^ and 37^+6^ weeks of gestation. Right ventricular end-diastolic volume (RVEDV) and right ventricular end-systolic volume (RVESV) were determined using the virtual organ computer-aided analysis software, and the parameters of right ventricular function were calculated.

**Results:**

Both RVEDV and RVESV were found to be significantly higher in fetuses with HLHS as compared to that in normal controls (*P* < 0.001). There were no significant differences in the parameters between fetuses with and without a visible left ventricular cavity (*P* > 0.05). Compared to fetuses with HLHS plus mild tricuspid regurgitation (TR), fetuses with HLHS plus severe TR exhibited lower right ventricular stroke volume (RVSV), right ventricular cardiac output (RVCO) and standardized RVCO (*P* < 0.05). The right ventricular ejection fraction (RVEF) was significantly lower in HLHS fetuses that had severe TR (*P* < 0.001).

**Conclusion:**

As the right ventricle is solely responsible for maintenance of circulation, the right ventricular systolic function undergoes compensatory enhancement in fetuses with HLHS and mild TR, compared to that in normal controls. Size of the left ventricle does not significantly affect the right ventricular function in HLHS. However, right ventricular systolic function may be impaired prenatally in HLHS fetuses that have severe TR.

## Background

The hypoplastic left heart syndrome encompasses a spectrum of cardiac malformations that are characterized by significant underdevelopment of the components of the left heart and the aorta, including the left ventricular cavity and mass [1]. Its main anatomical characteristics include mitral stenosis or atresia, aortic stenosis or atresia, and hypoplasia of left ventricle. The apex of the heart is usually formed by the right ventricle. The right ventricle is the single functional ventricle maintaining fetal circulation in these patients [2]. Generally, HLHS does not cause any adverse effect in utero. Most fetuses with HLHS could be delivered at term if no severe tricuspid regurgitation (TR) is present [3]. After birth, due to decreased pulmonary vascular resistance and closure of the ductus arteriosus, infants with HLHS develop a constellation of symptoms that include cyanosis, dyspnea, heart failure, hypoxemia and acidosis. Surgery is the only effective treatment. Therefore, evaluation of preoperative right ventricular function in fetuses with HLHS could provide valuable information for prenatal counseling and guide timely and effective clinical intervention after birth [4].

Four-dimensional spatio-temporal image correlation (STIC), which correlates the three-dimensional volume with cardiac phase, has been extensively used in screening and diagnosis of fetal cardiac defects [5–14]. However, STIC has not been widely used in the evaluation of fetal right ventricular function in HLHS. Unlike left ventricle, the right ventricle has a more irregular shape, and does not contract along its short axis. Therefore, traditional M-mode echocardiography is not an accurate method to assess its function. Using STIC technology in combination with the virtual organ computer-aided analysis (VOCAL) software, the right ventricular end-diastolic and systolic volumes (RVEDV and RVESV, respectively) can be more accurately measured.

In the present study, we used STIC in combination with VOCAL to compare the cardiac function parameters, such as stroke volume, cardiac output and ejection fraction, between fetuses with HLHS and gestational age-matched normal controls.

## Methods

### Study subjects

The study was approved by the Clinical Ethics Committee at the Second Xiangya Hospital of the Central South University in Changsha. All participating subjects were informed of the sensitivity, accuracy and limitations of the ultrasound examination. Written informed consent was obtained prior to enrollment.

Forty-nine pregnant women carrying fetuses with HLHS, and 180 pregnant women carrying normal fetuses were enrolled during routine prenatal ultrasound examinations conducted between September 2008 and October 2014. Only cases with situs solitus, atrio-ventricular concordance and ventriculo-arterial concordance were included in the study. Subjects with other cardiac comorbidities such as, transposition of great arteries, double outlet right ventricle and atrioventricular septal defect were excluded.

Severe TR was defined according to the following parameters: the maximum velocity of TR is above 2 m/s, with an area covered by the jet > 25 % of the atrium, reverse or absent blood flow during atrial systole in the ductus venous [15, 16].

### Instruments and methods

Voluson 730 Expert and Voluson E8 (GE Healthcare, USA) ultrasound systems were used with obstetric preset. The Doppler energy was set at < 100 mW/cm^2^. Fetal biometric measurements were performed on each scan. Subsequently, fetal echocardiography was performed by an expert and the STIC volume acquired.

Prior to STIC volume acquisition, fetal heart preset was selected and four-chamber view was displayed. The image was magnified with the heart occupying 1/3 to 1/2 of the screen. The gray scale image contrast was adjusted to afford a clear delineation of the four chambers of the fetal heart. After initial preparation, the STIC function was activated. Based on the size of fetal heart and the frequency of fetal movement, the acquisition time was set at 10–15 s and the scanning angle was set between 25 and 35°. The sample box included the whole fetal heart and its adjacent structures. Volume data was acquired when: 1) the probe was held still; 2) no fetal movement was present; 3) at least three complete cardiac cycles were included; 4) the fetus was in a supine position, and the angle between ultrasound beam and the heart was < 30°; and 5) There was no or very little shadowing from the ribs in the fetal heart. Qualified images were saved for off-line analysis.

4DView (versions 5.0–7.0, GE) was used to analyze the acquired volume data. In plane A, the fetal heart was repositioned in such a manner that the apex was in an upright position and the interventricular septum was perpendicular to the X axis. The X, Y, and Z axes were rotated to obtain the maximum visualization of the right ventricular cavity. Then select the image pseudo color for Sepia and SRI 5. After adjusting the brightness and contrast, the image was enlarged to 1.3 times and the cine speed reduced to 25 %. Since isovolumic contraction and relaxation time was short and had little impact on the ventricular volume, we defined end-diastole as the instant when the atrioventricular valve has just closed, and end-systole as the instant just prior to the opening of the atrioventricular valve.

VOCAL mode was used to estimate the volume of right ventricle. Since the endocardium of the right ventricle is rough and full of trabecular muscles, we traced the inner surface of ventricle manually. The rotation angle was set to 15^°^, and the reference point was placed in the right ventricle. After setting these parameters, the software automatically generated 12 images at different rotation angles for tracing. The endocardium was outlined on each image. The trabeculae and muscle bundles were considered as a part of the ventricular cavity. After the initial tracing was obtained, the curve was further trimmed in each plane to ensure accuracy. The software automatically calculated the RVEDV and RVESV. These measurements were performed in three consecutive cardiac cycles by the same examiner and the averages were used for further analysis.

The RVEDV and RVESV measurements were used to calculate the following parameters: right ventricular stroke volume (RVSV), right ventricular cardiac output (RVCO), right ventricular ejection fraction (RVEF), and RVCO/estimated fetal weight (EFW) [17]. In addition, RVCO/head circumference (HC), RVCO/abdominal circumference (AC) and RVCO/femur length (FL) were also calculated.

### Follow-up

Parents of the affected fetuses were informed of the findings and the potential outcomes. If the pregnancy was terminated, fetal autopsy was performed only after the written informed consent of the parents.

### Assessment of inter- and intra-examiner reproducibility

Inter-examiner and intra-examiner reproducibility was assessed separately by repeat examinations performed in a random sample of 30 cases each. For inter-examiner reproducibility, coefficient of variation was determined after re-examination by two examiners, while that for intra-examiner reproducibility was determined after re-examination by the same examiner 2 weeks after the initial examination.

### Statistical analysis

Statistical analysis was performed using SPSS 17.0 (Chicago, IL, SPSS Inc). All data are expressed as mean ± standard deviation (SD). Inter-group differences were assessed by Analysis of Covariance (ANCOVA), with gestational age considered as a covariate. A *P*-value < 0.05 was considered statistically significant.

## Results

### Follow-up data

A total of 49 cases of prenatal HLHS were enrolled. Parents of 42 cases opted for termination of pregnancy, while 4 fetuses died in utero. Diagnosis was confirmed on autopsy in all 46 cases. The remaining 3 cases were lost to follow-up.

### Success rate of STIC volume acquisition

**Fig. 1:**
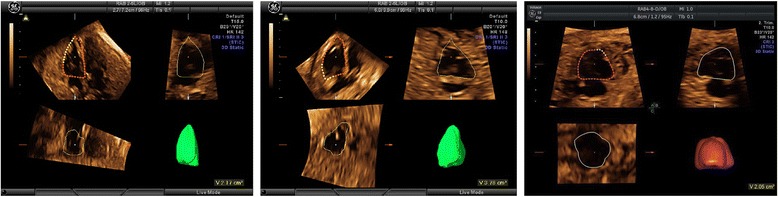
Measurement of right ventricular end-diastolic volume in a normal fetus **a** a fetus with HLHS and visible left ventricular cavity **b** and a fetus with HLHS and invisible left ventricular cavity **c**. *HLHS: Hypoplastic left heart syndrome*

**Fig. 2:**
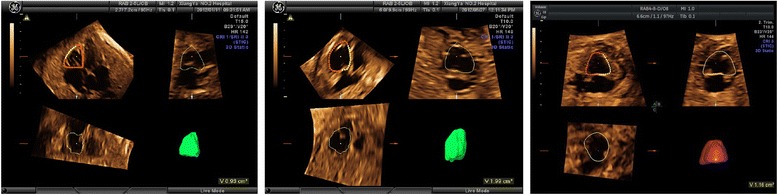
Measurement of right ventricular end-systolic volume in a normal fetus **a** a fetus with HLHS and visible left ventricular cavity **b** and a fetus with HLHS and invisible left ventricular cavity **c**. *HLHS: Hypoplastic left heart syndrome*

Among the 180 control fetuses, satisfactory STIC volume set was obtained in 151 cases (83.89 %): 55 cases between the 24^+0^ and 27^+6^ weeks, 51 cases between the 28^+0^ and 31^+6^ weeks, and 45 cases between the 32^+0^ and 37^+6^ weeks. Fetal RVEDV, RVESV and other right cardiac function parameters were calculated in these cases (Figs. 1a and 2a).

Out of the 46 cases with HLHS confirmed on autopsy, satisfactory STIC volume set was obtained in 39 cases (84.78 %): These included, 18 cases between the 24^+0^ and 27^+6^ weeks, 13 cases between the 28^+0^ and 31^+6^ weeks, and 8 cases between the 32^+0^ and 37^+6^ weeks. Fetal RVEDV, RVESV and other right cardiac function parameters were calculated in these cases (Fig. 1b, c and Fig. 2b, c).

### Fetuses with HLHS had larger right ventricular volume compared with control fetuses

Compared with the gestational age-matched normal fetuses, fetuses with HLHS had significantly increased RVEDV and RVESV (*P* < 0.001). No significant difference in terms of cardiac function parameters were observed between those with (*N* = 26) and without (*N* = 13) visible left ventricular cavity (*P* > 0.05) (Tables 1 and 2).

**Table 1:** Right ventricular volume and cardiac function parameters: Comparison between affected fetuses with and without visible left ventricular cavity, and gestational age-matched controls

Group	*N*	Gestational age (weeks)	RVEDV (mL)	RVESV (mL)	RVSV (mL)	RVCO (mL/min)	RVEF (%)
Control	151	30.16 ± 3.85	3.54 ± 1.50	1.33 ± 0.56	2.21 ± 0.97	321.89 ± 142.02	62.31 ± 4.15
HLHS-LV cavity visible	26	29.12 ± 3.54	4.18 ± 1.52^*^	1.88 ± 0.99^*^	2.30 ± 0.80^*^	337.89 ± 113.65^*^	56.74 ± 11.05^*^
HLHS-LV cavity invisible	13	29.50 ± 3.20	4.55 ± 1.39^*^	1.93 ± 0.70^*^	2.62 ± 0.83^*^	377.46 ± 124.96^*^	57.38 ± 6.99^*^

*Abbreviations*: *HLHS* Hypoplastic left heart syndrome, *LV* left ventricle, *RVEDV* right ventricular end-diastolic volume, *RVESV* right ventricular end-systolic volume, *RVSV* right ventricular stroke volume, *RVCO* right ventricular cardiac output, *RVEF* right ventricular ejection fraction

* *P* < 0.001, vs. control group, after adjusting for gestational age

**Table 2:** Estimated fetal weight and biological parameters: Comparison between the affected fetuses with and without visible left ventricular cavity, and gestational age-matched controls

Group	*N*	Gestational age (weeks)	RVCO/EFW (mL/min/kg)	RVCO/HC (mL/min/cm)	RVCO/AC (mL/min/cm)	RVCO/FL (mL/min/cm)
Control	151	30.16 ± 3.85	207.01 ± 31.59	11.30 ± 4.02	11.92 ± 3.86	54.84 ± 18.43
HLHS-LV cavity visible	26	29.12 ± 3.54	260.23 ± 68.07^*^	12.52 ± 3.36^*^	13.48 ± 3.73^*^	61.83 ± 16.23^*^
HLHS-LV cavity invisible	13	29.50 ± 3.20	276.71 ± 66.09^*^	13.86 ± 3.66^*^	14.61 ± 3.63^*^	67.18 ± 15.85^*^

*Abbreviations*: *HLHS* hypoplastic left heart syndrome, *LV* left ventricle, *RVCO* right ventricular cardiac output, *EFW* estimated fetal weight, *HC* head circumference, *AC* abdominal circumference, *FL* femur length

*P < 0.001, vs. control group, after adjusting for gestational age

### Fetuses with HLHS and severe TR had impaired right ventricular contractility

Compared with fetuses with HLHS and mild TR (*N* = 29), those with severe TR (*N* = 10) had significantly increased RVEDV (*P* < 0.05) and RVESV (*P* < 0.001), but lower RVSV, RVCO, standardized RVCO (RVCO/EFW, RVCO/HC, RVCO/AC, RVCO/FL) (*P* < 0.05) and RVEF (*P* < 0.001). No significant difference in RVEF was observed between fetuses with HLHS plus mild TR and normal controls (*P* > 0.05) (Table 3 and 4). These findings suggest that the fetuses with HLHS plus severe TR could have impaired right ventricular contractility.

**Table 3:** Right ventricular volume and fetal cardiac function parameters: Comparison between the affected fetuses with different degrees of tricuspid regurgitation and gestational age-matched controls

Group	*N*	Gestational age (weeks)	RVEDV (mL)	RVESV (mL)	RVSV (mL)	RVCO (mL/min)	RVEF (%)
Control	151	30.16 ± 3.85	3.54 ± 1.50	1.33 ± 0.56	2.21 ± 0.97	321.89 ± 142.02	62.31 ± 4.15
HLHS-mild TR	29	29.43 ± 3.64	4.17 ± 1.43^*^	1.62 ± 0.67^*^	2.55 ± 0.81^*^	369.35 ± 118.41^*^	61.92 ± 4.78
HLHS-severe TR	10	28.61 ± 2.60	4.69 ± 1.59^*#^ (*P* = 0.008)	2.71 ± 0.99^*##^	1.98 ± 0.66^#^ (*P* = 0.003)	298.09 ± 101.94^#^ (*P* = 0.014)	42.56 ± 4.93^*##^

*Abbreviations*: *HLHS* hypoplastic left heart syndrome, *TR* tricuspid regurgitation, *RVEDV* right ventricular end-diastolic volume; *RVESV* right ventricular end-systolic volume, *RVSV* right ventricular stroke volume, *RVCO* right ventricular cardiac output, *RVEF* right ventricular ejection fraction

* P < 0.001 vs. control group, after adjusting for gestational age; ^#^ *P <* 0.05, ^##^ *P <* 0.001 vs. HLHS-mild TR, after adjusting for gestational age

**Table 4:** Estimated fetal weight and biological parameters: Comparison between the affected fetuses with different degrees of tricuspid regurgitation and gestational age-matched controls

Group	*N*	Gestational age (weeks)	RVCO/EFW (mL/min/kg)	RVCO/HC (mL/min/cm)	RVCO/AC (mL/min/cm)	RVCO/FL (mL/min/cm)
Control	151	30.16 ± 3.85	207.01 ± 31.59	11.30 ± 4.02	11.92 ± 3.86	54.84 ± 18.43
HLHS-mild TR	29	29.43 ± 3.64	280.00 ± 63.62^*^	13.70 ± 3.32^*^	14.62 ± 3.58^*^	63.24 ± 13.90^*^
HLHS-severe TR	10	28.61 ± 2.60	224.32 ± 61.64^#^ (*P* = 0.013)	10.85 ± 3.16^#^ (*P* = 0.006)	11.66 ± 3.23^#^ (*P* = 0.015)	53.59 ± 15.66^#^ (*P* = 0.008)

*Abbreviations*: *HLHS* hypoplastic left heart syndrome, *TR* tricuspid regurgitation, *RVCO* right ventricular cardiac output, *EFW* estimated fetal weight, *HC* head circumference, *AC* abdominal circumference, *FL* femur length

* *P <* 0.001 vs. control group, after adjusting for gestational age; ^#^ *P <* 0.05 vs. HLHS-mild TR, after adjusting for gestational age

### Inter- and intra- examiner reproducibility

The inter-examiner reproducibility was 92.5–88.2 %, while the intra-examiner reproducibility was 93.4–89.1 %.

## Discussion

In the present study, a total of 226 fetuses were initially included. However, data acquisition and analysis was not successful in all cases due to poor fetal positioning (*N* = 12), frequent fetal movement (*N* = 9), and low image quality (*N* = 15). As a result, a total of 190 fetuses with acceptable STIC images (fetuses with HLHS [*N* = 39]; normal controls (*N* = 151), were studied. The overall success rate of data collection in the study was 84.07 %. Our study suggests that measurements of fetal ventricular volume in the different cardiac phases can be done with a high feasibility using STIC technology, which is an available tool for obtaining data on fetal cardiac volume throughout the cardiac cycle, and assess fetal cardiac function under both physiological and pathological conditions.

Fetuses with HLHS have a left ventricle with either a very poor function or no function at all, due to which sufficient amount of blood cannot be pumped into the aorta. This effectively renders the right ventricle as the only functional ventricle for maintenance of the systemic and fetal pulmonary circulation [18, 19]. Surgical interventions, including heart transplant and multi-stage surgery such as Norwood procedure, are the only effective treatment for HLHS. Norwood procedure has three stages which allow the pulmonary vascular bed to gradually adapt to the increased blood volume, and eventually a complete transition to separate pulmonary circulation is achieved after Fontan procedure. Since the right ventricle will function as the systemic ventricle and will pump the blood to the body, right ventricular dysfunction is a contraindication for surgical treatment of HLHS [18].

In the present study, fetuses with HLHS had a significantly increased RVEDV and RVESV as compared to that in their gestational age-matched controls, which may represent a compensatory mechanism. Further, no difference in RVEF was observed between the affected fetuses with mild TR and the normal controls, which suggests that fetuses with HLHS plus mild TR tend to maintain a relatively normal right ventricular systolic function. Our study also suggests that the presence of hypoplasia or atresia of left ventricle and/or ascending aorta could severely impair the left ventriclar function, thus rendering the right ventricle as solely responsible for maintenance of circulation. Therefore, increased RVCO becomes necessary in fetuses with HLHS, only this can ensure that the fetal circulation meets the needs of growth and development of the fetus in utero, while the compensaory increase of right ventricular volume and compensatory enhancement of right ventricular systolic function in fetus with HLHS is to ensure that the appropriate increase of RVCO.

To understand whether the size of the left ventricle had any effect on the right ventricular function, we further divided the affected fetuses into two subgroups: HLHS with and without a visible left ventricular cavity. No significant differences were noted between these two subgroups in terms of right ventricular volume and cardiac function parameters, which indicates that the absence of left ventricle did not have any impact on the right ventricular function, which is consistent with the findings from other recent studies [3, 19]. For instance, Szwast et al. [3] assessed the fetal cardiac function by Doppler and concluded that negative interactions of a noncontributory left ventricular cavity on right ventricular performance were negligible in the fetus. Miller et al. [20] used Velocity Vector Imaging (VVI) technology to study the regional and global myocardial motion of right ventricle in fetuses with HLHS, and concluded that the left ventricular volume and absence of left ventricular cavity in fetuses with HLHS did not affect the right ventricular myocardial motion. However, whether the size of underdeveloped left ventricle could affect the right ventricular function in children with HLHS is still controversial [21, 22]. In a recent study by Wisler et al. [21], the size of the left ventricle in patients with HLHS had an impact on the right ventricular function only before Fontan procedure; and that it did not affect the right ventricular function either before Norwood and Glenn procedure, or after Fontan procedure. Considering the high risk of mortality, the high cost of surgical treatment and the unfavorable post-surgical prognosis, all parents in our study opted for termination of pregnancy after counseling. Therefore, we were unable to carry out postnatal follow up.

According to the available literature, severe TR rarely causes intrauterine death in HLHS. However, in fetuses that survive to term, severe TR places the children undergoing postnatal surgery at higher risk [3, 18, 23, 24]. To understand the clinical significance of TR on right ventricular function, we categorized the affected fetuses into two subgroups: HLHS with severe regurgitation and HLHS with mild regurgitation. The right ventricular volume and cardiac function parameters were compared between the two subgroups, as also with the gestational age-matched controls. Compared to the affected fetuses who had mild TR, those with severe TR had significantly greater RVEDV and RVESV, but lower RVSV, RVCO, standardized RVCO (RVCO/EFW, RVCO/HC, RVCO/AC, RVCO/FL) and RVEF. In addition, the RVEDV and RVESV in both the HLHS subgroups was greater than that in the normal controls. Further, fetuses with HLHS plus severe TR, but not those with mild TR, had lower RVEF than that observed in the controls. These findings suggest a significant impairment of right ventricular function in the affected fetuses. Our study also showed that the vast majority of HLHS fetuses had mild TR which could be comparable to the physiological TR observed in normal fetuses. The physiological TR may be a consequence of un-inflated lung, high pulmonary vascular resistance, and relatively high pulmonary artery pressure in utero. Mild TR usually does not affect the right ventricular function. As fetuses with HLHS have only one effectively functional ventricle (right ventricle), the RVEDV, RVESV, RVSV and RVCO tend to be significantly higher than that observed in the normal controls. According to Frank-Starling law of the heart, there is a positive correlation between myocardial contractility and end-diastolic volume. Increased RVEDV could improve cardiac contractility and contribute to the relatively normal fetal growth and development in HLHS. In our study, a small number of HLHS fetuses with severe TR, although exhibiting increased right ventricular end-diastolic volume, had poor cardiac function as compared to that in those fetuses with mild TR. Their RVEF was also lower compared to that of the normal controls. These findings suggested that the HLHS fetuses with severe TR had already impaired right ventricular contractility, which was probably due to the effective blood circulation decreasing significantly through the tricuspid valve because of severe regurgitation. According to the Frank-Starling curve, the right ventricular systolic dysfunction would ultimately occur as the right ventricular preload increased beyond a certain extent.

For affected fetuses with a narrow foramen ovale or an intact atrial septum, there are few cardiac function parameters other than pulmonary vein Doppler indices that can be used to guide prenatal counseling and inform postnatal management of single ventricular circulation [25]. Since cardiac dysfunction remains an important risk factor for perioperative mortality and is known to reduce the long-term survival after surgery [26], a precise evaluation of the right ventricular function of fetuses with HLHS is particularly important. In this study, the changes of right ventricular function in fetal HLHS were confirmed by STIC technology, which provides a useful information for the clinical perinatal treatment.

### Limitations of the study

The main limitation of this study is that there is no postnatal follow-up to test the fetal findings as parents chose to terminate the pregnancy. Poor image quality was one of the factors that could have affected the data analysis, as the STIC technology requires a high quality volume set. Further, the image acquisition process is liable to be affected by a variety of factors, including fetal size, fetal position, fetal movements, fetal rib shadowing, amniotic fluid volume and by maternal factors. Owing to the relative rarity of cases, the small sample size is another factor, which may have reduced the power of the statistical analysis. Thus, further studies with larger sample sizes are warranted.

## Conclusion

The size of the left ventricular cavity in fetuses with HLHS does not significantly affect the right ventricular function. Fetuses with HLHS and mild TR had enhanced right ventricular systolic function compared to normal controls, which probably represents a compensatory mechanism. However, those with severe TR are likely to have impaired right ventricular systolic function in utero, which requires a prompt clinical intervention.
